# A comparison of airway pressures for inflation fixation of developing mouse lungs for stereological analyses

**DOI:** 10.1007/s00418-020-01951-0

**Published:** 2020-12-29

**Authors:** David Pérez-Bravo, Despoina Myti, Ivana Mižíková, Tilman Pfeffer, David E. Surate Solaligue, Claudio Nardiello, István Vadász, Susanne Herold, Werner Seeger, Katrin Ahlbrecht, Rory E. Morty

**Affiliations:** 1 Department of Lung Development and Remodelling, Max Planck Institute for Heart and Lung Research, member of the German Center for Lung Research (DZL), Parkstrasse 1, 60231 Bad Nauheim, Germany; 2 Department of Internal Medicine (Pulmonology), University of Giessen and Marburg Lung Center (UGMLC), member of the German Center for Lung Research (DZL), Aulweg 123, 35394 Giessen, Germany; 3 Regenerative Medicine Program, The Ottawa Hospital Research Institute, 501 Smyth (Box 511), Ottawa, ON 1H 8L6 Canada; 4 Department of Cellular and Molecular Medicine, University of Ottawa, 451 Smyth Road, Ottawa, ON K1H 8M5 Canada; 5 Centre for Paediatric and Adolescent Medicine, Heidelberg University Hospital, Im Neuenheimer Feld 430, 69120 Heidelberg, Germany; 6 Our Lady’s Hospital, MoathillCo. Meath, Navan, C15 RK7Y Ireland; 7 Cardio Pulmonary Institute, Justus Liebig University Giessen, Klinikstrasse 33, Giessen, Germany; 8 Institute for Lung Health (ILH), Justus Liebig University Giessen, Aulweg 130, Giessen, Germany

**Keywords:** Airway pressure, Inflation fixation, Mouse, Lung volume, Lung development, Stereology

## Abstract

The morphometric analysis of lung structure using the principles of stereology has emerged as a powerful tool to describe the structural changes in lung architecture that accompany the development of lung disease that is experimentally modelled in adult mice. These stereological principles are now being applied to the study of the evolution of the lung architecture over the course of prenatal and postnatal lung development in mouse neonates and adolescents. The immature lung is structurally and functionally distinct from the adult lung, and has a smaller volume than does the adult lung. These differences have raised concerns about whether the inflation fixation of neonatal mouse lungs with the airway pressure (*P*_aw_) used for the inflation fixation of adult mouse lungs may cause distortion of the neonatal mouse lung structure, leading to the generation of artefacts in subsequent analyses. The objective of this study was to examine the impact of a *P*_aw_ of 10, 20 and 30 cmH_2_O on the estimation of lung volumes and stereologically assessed parameters that describe the lung structure in developing mouse lungs. The data presented demonstrate that low *P*_aw_ (10 cmH_2_O) leads to heterogeneity in the unfolding of alveolar structures within the lungs, and that high *P*_aw_ (30 cmH_2_O) leads to an overestimation of the lung volume, and thus, affects the estimation of volume-dependent parameters, such as total alveoli number and gas-exchange surface area. Thus, these data support the use of a *P*_aw_ of 20 cmH_2_O for inflation fixation in morphometric studies on neonatal mouse lungs.

**Supplementary Information:**

The online version contains supplementary material available at 10.1007/s00418-020-01951-0.

## Introduction

Lung organogenesis occurs over a protracted period in mammals that starts in the early embryonic period, and continues into adolescence (Schittny 2017). Over the course of lung organogenesis, marked changes occur in the cellular and extracellular composition (Whitsett et al. 2019) and mechanical properties and architecture (Schittny 2017) of the lungs. The study of structural changes that occur during lung development rely on quantitative information about the lung structure, such as morphometric stereological methods (Weibel et al. 2007) that permit estimation of the density and number of alveoli, the gas-exchange surface area, and septal thickness, amongst other parameters.

The reliability of these estimations is critically dependent upon proper inflation of lungs during embedding and sectioning (Hsia et al. 2010). The airway pressure, *P*_aw_, determines the extent of lung inflation, including the unfolding of alveolar structures within the lungs. Thus, selection of appropriate *P*_aw_ is essential, as an optimal *P*_aw_ will expand—but not distend—the lungs, and will unfold the alveolar structures within the lungs such that reliable and reproducible quantitative measurements are possible (Hsia et al. 2010). The plethora of studies [for example (Limjunyawong et al. 2015; Schulte et al. 2019)] that quantify elements of adult mouse lung structure have established 20–25 cmH_2_O as an appropriate *P*_aw_ for intratracheal instillation of fixative solutions. This approach is widely used to study the microscopic structure of adult healthy and diseased mouse lungs.

Increasing interest in the structural changes that occur during normal and abnormal development of immature lungs [reviewed in (Lignelli et al. 2019)] has stimulated efforts to quantify elements of the lung structure in newborn or immature, developing mice. Newborn and developing mouse lungs are structurally and functionally distinct from adult lungs, where, pronounced changes in the cellular and extracellular components of the lungs accompany lung maturation. Compared to adult lungs, the more cellular nature of developing septa (Ruiz-Camp et al. 2019) and less mature extracellular matrix (ECM) network (Mižíková and Morty 2015) in newborn lungs affect the compliance of the lungs and may render lungs more or less sensitive to distension by *P*_aw_. Additionally, the small size, larger tissue-to-air volume ratio, and high surface-to-volume ratio of developing mouse lungs might limit the ability of instilled fixative to properly enter the lung.

For these reasons, a side-by-side comparison of a *P*_aw_ of 10, 20, and 30 cmH_2_O was performed, where immature, developing mouse lungs were inflated at one of these three *P*_aw_, and then subjected to lung volume estimation and stereological analysis. The data presented herein demonstrate that a comparatively low inflation fixation *P*_aw_ of 10 cmH_2_O results in heterogeneous unfolding of alveolar structures within the lungs, which impacts stereologically determined parameters to describe the lung structure. Similarly, a comparatively high inflation fixation *P*_aw_ of 30 cmH_2_O confounds estimation of lung volume, and hence, affects the reliability of volume-dependent stereologically determined parameters. Collectively, these data support the use of an inflation fixation *P*_aw_ of 20 cmH_2_O in stereology studies of immature, developing mouse lungs.

## Materials and methods

### Animal studies

Animal procedures were conducted in accordance with local and national regulations. Newborn C57BL/6J mouse pups (Charles River, Sülzfeld, Germany) were maintained to postnatal day (P)14. Sex of the mouse pups was determined by visualization of external genitalia (Wolterink-Donselaar et al. 2009). Dams and pups were maintained on a 12-h/12-h light–dark cycle, and received food and water ad libitum.

### Animal studies

**Fig. 1:**
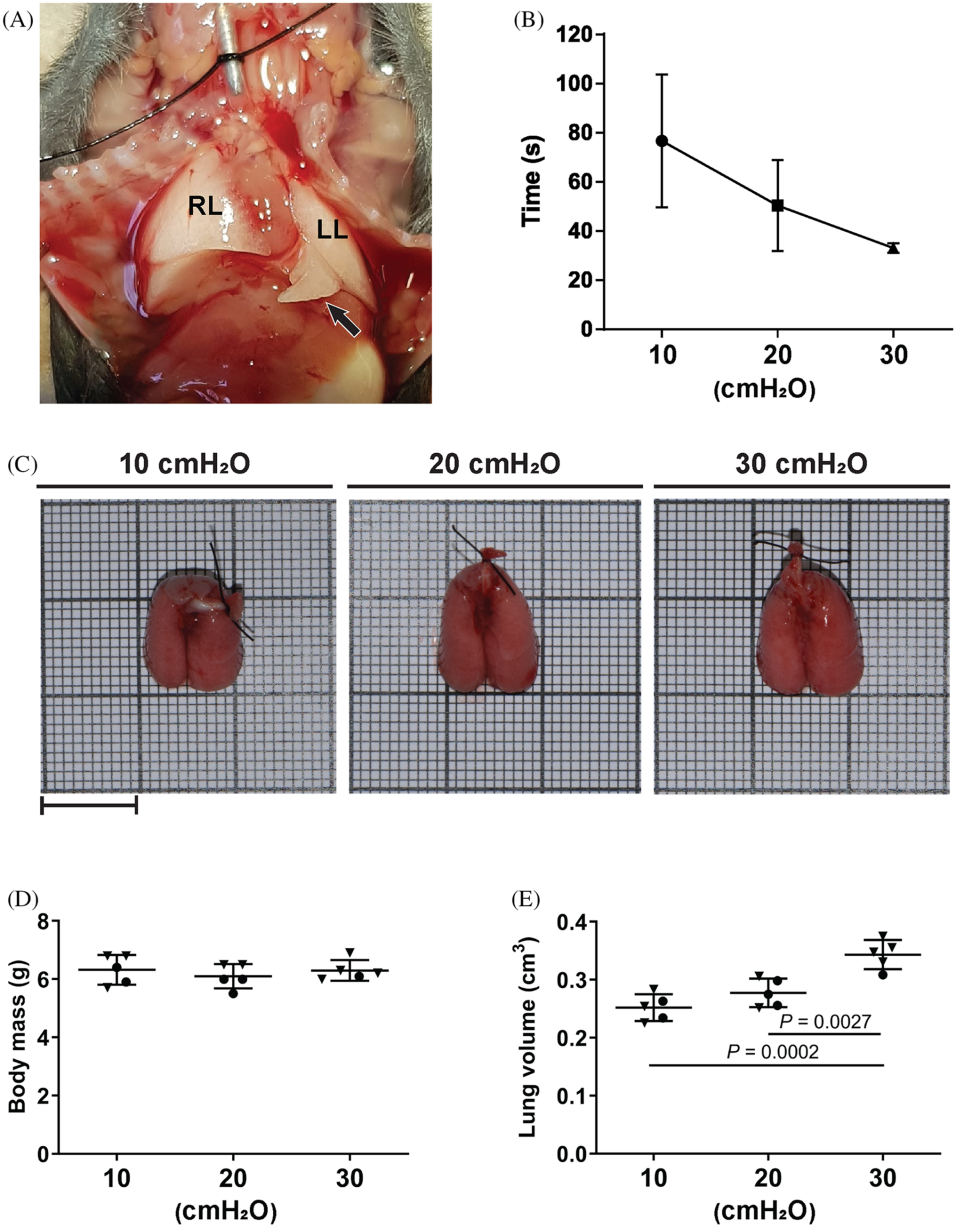
Lung inflation and volume at variable airway pressures. **a** Lung inflation was considered complete when the cardiac lobe was fully inflated, and the most distal aspect of the cardiac lobe (arrow) projected maximally outwards. **b** The time required for maximum inflation of lungs with fixative at airway pressures (*P*_aw_) of 10 cmH_2_O, 20 cmH_2_O, and 30 cmH_2_O. **c** Gross morphology of mouse lungs harvested at postnatal day 14 and inflated with fixative at *P*_aw_ of 10, 20, and 30 cmH_2_O; superimposed on 1-mm graph paper. Images are representative of the trends overserved in four other lungs per experimental group. Scale bar: 1 cm. The **d** mouse body mass was measured, and **e** lung volume was estimated using Cavalieri’s principle. Data reflect mean ± S.D. (*n* = 5 lungs per experimental group). Closed inverted triangles denote female animals, closed circles denote male animals. Data comparisons were performed by one-way ANOVA with Tukey’s post hoc test, with *P* values < 0.05 indicated. *LL* left lung, *RL* right lung

Mouse pups were killed by administration of 500 mg.kg^−1^ sodium pentobarbital (Narcoren; Bohringer, Ingelheim, Germany), via the intraperitoneal route. The thoracic cavity was exposed by midsternal thoracotomy and dissection of the diaphragm at the point of contact with the ribcage. Tracheostomy was undertaken with a 22G blunt-ended cannula (CML Supply, Lexington, U.S.A.), which was fixed in place with a USP 4/0 non-absorbable surgical suture (Supramid, St. Vith, Belgium), as depicted in Fig. 1a. The lungs were fixed by intratracheal instillation of 1.5% (m/v) paraformaldehyde, 1.5% (m/v) glutaraldehyde in 150 mM HEPES, pH 7.4. Three groups of five mouse pups each were used to compare, side-by-side, the impact of different *P*_aw_ on the estimation of elements of the lung structure by design-based stereology. To this end, lungs were inflated with fixative at *P*_aw_ of 10, 20 and 30 cmH_2_O, and took between 35 and 75 s to inflate (Fig. 1b), depending on *P*_aw_. Lungs were considered fully inflated once no visible increase in lung expansion was noted, and when the cardiac lobe was sufficiently turgid to maximally project outwards from the thorax (Fig. 1a). After inflation, the tracheostomy needle was partially withdrawn, and the trachea was ligated with a suture as described above, below the placement of the tracheostomy suture. The lungs, heart, and thymus were removed *en bloc*, and placed in the same fixative solution for 24 h at 4 °C in a 5 ml Eppendorf tube. Lungs were then dissected free of the heart and thymus and photographed on a background of 1-mm graph paper to record the gross anatomy (Fig. 1c).

Lung tissue was collected according to systematic uniform random sampling (Tschanz et al. 2014): Lungs were embedded in agarose [in 2% (m/v) agar–agar], and agar blocks were sectioned into 3-mm slabs. Lung volume was estimated using Cavalieri’s principle (Tschanz et al. 2011). Lung tissue was transferred from agar slabs into 15 ml glass vials, and treated with 0.1 M sodium cacodylate (Serva, Heidelberg, Germany; 15540.03), 1% (m/v) osmium tetroxide (Roth, Karlsruhe, Germany; 8371.3), and 2.5% (m/v) uranyl acetate (Serva, Darmstadt, Germany; 77870.01) in ddH_2_O. Lung tissue was embedded in glycol methacrylate resin (Technovit 7100; Heareus Kulzer, Hanau, Germany; 64709003), which hardened over 48 h (Mühlfeld et al. 2013). Technovit blocks were sectioned at 2 µm. For estimation of total number of alveoli and alveolar density, every first and third section of a consecutive series of sections was collected and stained with Richardson’s stain (Fehrenbach et al. 2008; Knust et al. 2009). For the analysis of additional parameters, four sections, representing every 10th section of a consecutive series in the same block, were collected and stained with Richardson’s stain (Schneider and Ochs 2013). A NanoZoomer-XR C12000 Digital slide scanner (Hamamatsu Photomics Deutschland, Herrsching am Ammersee, Germany) collected digital images of tissue sections.

### Design-based stereology

Analysis of lung structure was performed using the principles of design-based stereology that have been extensively reviewed for adult mouse lungs (Mühlfeld and Ochs 2013; Ochs and Mühlfeld 2013; Mühlfeld et al. 2015), in accordance with American Thoracic Society/European Respiratory Society guidelines for quantitative assessment of lung structure (Hsia et al. 2010), described in detail previously (Pozarska et al. 2017; Schneider et al. 2017; Wagener et al. 2020). For the estimation of alveolar density and alveolar number, one pair of sections was selected for analysis at a 3 and 10% coverage of the regions of interest of each section. For estimation of surface density, gas-exchange surface area, arithmetic mean septal thickness, and mean linear intercept (MLI), four sections per tissue block were selected for analysis, at 3 and 6% coverage of the regions of interest of each section; except for estimation of parenchymal volume, where a 10% coverage of the region of interest of each section was used.

### Statistics and stereological precision

Data are presented as mean ± SD. Differences were assessed by one-way ANOVA with Tukey’s post hoc test for multiple comparisons. *P* values ˂0.05 were regarded as significant. Statistical analyses were performed with GraphPad Prism v. 8.4.2. The presence of statistical outliers was tested by Grubbs’ test, and none were found. The coefficient of error (CE), the coefficient of variation (CV) and the quotient CE^2^/CV^2^ were calculated for each stereological parameter, where a CE^2^/CV^2^ < 0.5 validated the precision of the measurements (Tables 1 and 2). To describe the variance within hydrostatic *P*_aw_ groups comparing 3% *versus* 10% (for alveoli number and alveolar density) or 3% *versus* 6% (for all other stereology parameters) coverage of the regions of interest, the absolute value of the difference between the two mean values of interest was calculated.

**Table 1:** Stereological parameters for 14-day old mouse lungs that were inflated with fixative solution at airway pressures of 10, 20, and 30 cmH_2_O at a 3% coverage of the regions of interest for all stereological parameters

			10 cmH_2_O			20 cmH_2_O				30 cmH_2_O				
			Mean ± S.D			Mean ± S.D			*P* value *vs*. 10 cmH_2_O	Mean ± S.D			*P* value *vs*. 10 cmH_2_O	*P* value *vs*. 20 cmH_2_O
*V* (lung) [cm^3^]			0.252 ± 0.023			0.277 ± 0.024			0.2636	0.343 ± 0.025			0.0002	0.0027
CE	CV	CE^2^/CV^2^	0.040819	0.091275	0.199996	0.039587	0.088520	0.199996		0.032842	0.073436	0.200005		
*V*_V_ (par/lung) [%]			93.860 ± 1.365			90.615 ± 4.189			0.2818	92.460 ± 2.518			0.7896	0.6254
CE	CV	CE^2^/CV^2^	0.006504	0.014543	0.200001	0.020673	0.046226	0.200002		0.012180	0.027235	0.200005		
*N* (alv, lung) × 10^6^			4.527 ± 0.559			4.565 ± 0.481			0.9939	5.610 ± 0.276			0.0107	0.0130
CE	CV	CE^2^/CV^2^	0.0552257	0.091274	0.199998	0.039587	0.088519	0.200001		0.032841	0.073436	0.199993		
*N*_V_ (alv/par) × 10^6^ [cm^−3^]			19.298 ± 0.261			18.193 ± 0.072			0.5504	17.745 ± 0.074			0.3231	0.9012
CE	CV	CE^2^/CV^2^	0.060454	0.135179	0.200001	0.017750	0.039690	0.200002		0.018655	0.041715	0.199989		
*S* (alv epi, lung) [cm^2^]			190.336 ± 14.435			219.982 ± 25.870			0.0959	265.563 ± 19.464			0.0002	0.0109
CE	CV	CE^2^/CV^2^	0.033917	0.075842	0.199993	0.052593	0.117602	0.199998		0.032777	0.073293	0.199992		
*S*_V_ (Alv epi/par) [cm^−1^]			808.912 ± 67.938			877.295 ± 63.344			0.3003	841.383 ± 76.276			0.7452	0.6993
CE	CV	CE^2^/CV^2^	0.037560	0.083987	0.199999	0.032290	0.072203	0.199998		0.040542	0.090655	0.199999		
*τ* (sep) [µm]			8.396 ± 0.567			6.379 ± 0.935			0.0015	5.963 ± 0.960			0.0003	0.6129
CE	CV	CE^2^/CV^2^	0.030225	0.067586	0.199995	0.027698	0.061936	0.199991		0.072010	0.161019	0.200001		
MLI [µm]			32.927 ± 3.156			33.025 ± 3.426			0.9987	35.938 ± 2.774			0.3163	0.3383
CE	CV	CE^2^/CV^2^	0.042874	0.095870	0.199997	0.046400	0.103753	0.200002		0.034525	0.077200	0.200001		

*alv* alveoli; *alv air* alveolar airspaces; *alv epi* alveolar epithelium; *CE* coefficient of error; *CV* coefficient of variation; *MLI* mean linear intercept; *N* number; *N*_*V*_ numerical density; *par*, parenchyma; *S* surface area; *S*_*V*_ surface density; *τ* arithmetic mean septal thickness; *V* volume; *V*_*V*_ volume density. Values are presented as mean ± S.D. (*n* = 5 lungs per group). Data comparisons were made by one-way ANOVA with Tukey’s post hoc test

**Table 2:** Stereological parameters for 14-day old mouse lungs that were inflated with fixative solution at airway pressures of 10, 20, and 30 cmH_2_O at a 10% (for alveoli number and alveolar density) and 6% (for all other stereology parameters) coverage of the regions of interest

			10 cmH_2_O			20 cmH_2_O				30 cmH_2_O				
			Mean ± S.D			Mean ± S.D			*P* value *vs*. 10 cmH_2_O	Mean ± S.D			*P* value *vs*. 10 cmH_2_O	*P* value *vs*. 20 cmH_2_O
*V* (lung) [cm^3^]			0.252 ± 0.023			0.277 ± 0.024			0.2636	0.343 ± 0.025			0.0002	0.0027
CE	CV	CE^2^/CV^2^	0.040819	0.091275	0.199996	0.039587	0.088520	0.199996		0.032842	0.073436	0.200005		
*V*_*V*_ (par/lung) [%]			93.860 ± 1.365			90.615 ± 4.189			0.2818	92.460 ± 2.518			0.7896	0.6254
CE	CV	CE^2^/CV^2^	0.006504	0.014543	0.200011	0.020673	0.046226	0.200002		0.012180	0.027235	0.200005		
*N* (alv, lung) × 10^6^			4.783 ± 0.530			4.488 ± 0.517			0.6054	5.384 ± 0.369			0.1569	0.0294
CE	CV	CE^2^/CV^2^	0.049555	0.110808	0.200001	0.051560	0.115291	0.200002		0.030644	0.068521	0.200006		
*N*_*V*_ (alv/par) × 10^6^ [cm^−3^]			20.236 ± 1.458			17.848 ± 0.658			0.0043	16.973 ± 0.253			0.0004	0.3348
CE	CV	CE^2^/CV^2^	0.032224	0.072055	0.200000	0.016497	0.0368880	0.200004		0.006669	0.014912	0.200009		
*S* (alv epi, lung) [cm^2^]			210.882 ± 21.608			214.124 ± 21.952			0.8922	246.997 ± 17.529			0.0692	0.1487
CE	CV	CE^2^/CV^2^	0.045823	0.102464	0.199997	0.045849	0.102522	0.199998		0.031739	0.070970	0.200003		
*S*_*V*_ (Alv epi/par) [cm^−1^]			892.535 ± 67.139			853.241 ± 43.026			0.5329	780.202 ± 56.886			0.0215	0.1445
CE	CV	CE^2^/CV^2^	0.033641	0.075223	0.200003	0.022552	0.050427	0.200006		0.032607	0.072912	0.199997		
*τ* (sep) [µm]			7.601 ± 0.418			6.449 ± 0.176			0.0314	6.416 ± 0.977			0.0271	0.9962
CE	CV	CE^2^/CV^2^	0.024580	0.054963	0.199997	0.012186	0.027250	0.199981		0.068077	0.152224	0.200002		
MLI [µm]			29.818 ± 2.758			34.078 ± 2.590			0.0511	38.670 ± 2.234			0.0004	0.0354
CE	CV	CE^2^/CV^2^	0.041359	0.092481	0.200002	0.033985	0.075992	0.200004		0.025838	0.057775	0.2000004		

*alv* alveoli; *alv air* alveolar airspaces; *alv epi* alveolar epithelium; *CE* coefficient of error; *CV* coefficient of variation; *MLI* mean linear intercept; *N* number; *N*_*V*_ numerical density; *par* parenchyma; *S* surface area; *S*_*V*_ surface density; *τ* arithmetic mean septal thickness; *V* volume; *V*_*V*_ volume density. Values are presented as mean ± S.D. (*n* = 5 lungs per group). Data comparisons were made by one-way ANOVA with Tukey’s post hoc test

## Results and discussion

For the stereological analysis of distal lung structure, lungs from experimental animals can be fixed by intratracheal instillation of fixative (Mühlfeld and Ochs 2013; Ochs and Mühlfeld 2013). The objective of the present study was to determine whether a *P*_aw_ of 20 cmH_2_O for inflation fixation—the *P*_aw_ employed in studies with mature, adult mouse lungs—is also suitable for the stereological analysis of immature, developing mouse lungs.

This is important, since in the words of Drs. Wheeler, Wing and Zingarelli: “Children are not small adults!” (Wheeler et al. 2011). The compliance of the respiratory system diminishes during development, concomitant with the evolution of mechanical properties of the parenchyma and the airways (Sly et al. 2005). The lungs of children and adolescents have fewer alveoli compared to adults, which increase in number during early life and adolescence (Herring et al. 2014), to ≈480 million alveoli in adults (Ochs et al. 2004). The alveoli are smaller in infants, and are approximately double in size in adult lungs (Zeman and Bennett 2006). Parallel trends in increasing alveoli number and decreasing alveoli size have also been noted during postnatal growth in mice (Pozarska et al. 2017) and rats (Tschanz et al. 2014). Pertinent to the present study, at the widely employed P14 endpoint in studies on normal and abnormal postnatal lung development in mice (Nardiello et al. 2017), neither the progressively increasing alveolar density, nor the progressively decreasing mean alveolar volumes, have plateaued out at their respective adult values (Pozarska et al. 2017). Thus, it was a matter of concern that the different structural and functional properties of immature mouse lungs may result in tissue distortion by *P*_aw_ conventionally used for instillation-fixation of adult mouse lungs.

This concern has already been raised in studies on human lung disease modeled in mice that are characterized by loss of parenchymal tissue, such as modeling of emphysema in adult mice. In those studies, the airspaces of a disease lung with reduced elastic recoil will exhibit increased expansion over those in healthy lungs, which would stretch the alveolar septa. Thus, information gleaned about airspace enlargement and septal thickness would be confounded by the degree of inflation (Mühlfeld and Ochs 2013). Indeed, it has been noted that the estimation of the MLI strongly depends on the inflation status of the lung, where suboptimal inflation represents a key pitfall of the MLI parameter (Knudsen et al. 2010; Mühlfeld et al. 2015). As such, the *P*_aw_ used for inflation fixation in mature, adult mouse lungs may not be suitable for the inflation fixation of immature, developing mouse lungs, given the different mechanical properties of the immature lung tissue.

A spectrum of *P*_aw_ are routinely employed for the instillation fixation of adult mouse lungs, including 20 cmH_2_O (Schulte et al. 2019) and 25 cmH_2_O (Limjunyawong et al. 2015) which represent the recommended pressure range (20–25 cmH_2_O) for fixative instillation postmortem in adult mouse lungs (Hsia et al. 2010). In the present study, three different *P*_aw_ were selected for instillation fixation of P14 mouse lungs with HEPES-buffered paraformaldehyde/glutaraldehyde: 10 cmH_2_O, 20 cmH_2_O, and 30 cmH_2_O (Fig. 1c). Inflation of the lungs was considered complete when the lungs were no longer visibly inflating, and when the cardiac lobe was sufficiently turgid to maximally project outwards from the thorax (Fig. 1a). Using these criteria, the time for complete inflation was longest (≈ 75 s) using 10 cmH_2_O *P*_aw_, and shortest (≈ 35 s) using 30 cmH_2_O (Fig. 1b).

Mice of comparable body masses (6.2 ± 0.4 g) were employed for these studies (Fig. 1d), given that lung volume may depend on body mass (Pozarska et al. 2017). As such, all 15 mice employed in the study were expected to exhibit similar lung volumes. The lung volumes estimated for the 10 cmH_2_O (0.25 ± 0.023 g) and 20 cmH_2_O (0.27 ± 0.025 g) groups were comparable (Fig. 1e). However, the 30 cmH_2_O group exhibited higher lung volumes (0.34 ± 0.025 g; Fig. 1e). These data suggested that the higher *P*_aw_ of 30 cmH_2_O may have overinflated the lungs.

**Fig. 2:**
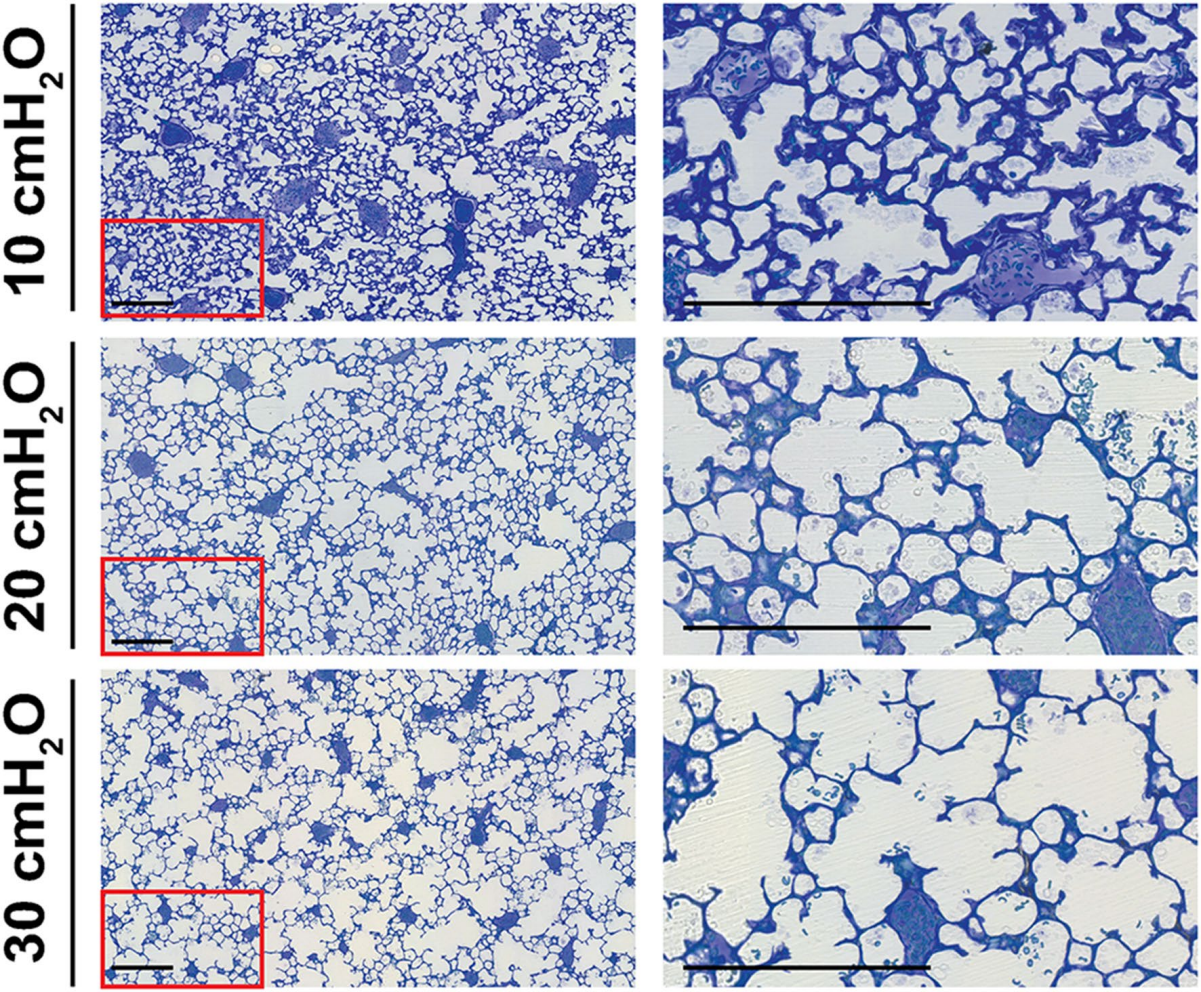
Distal lung structure at variable airway pressures. Distal lung structure was visualized in plastic-embedded lung tissue stained with Richardson’s stain, after lung inflation with fixative at airway pressures of 10, 20, and 30 cmH_2_O. The area demarcated with a red frame in the images *at left* are presented at higher magnification *at right*. Scale bar: 200 µm

**Fig. 3:**
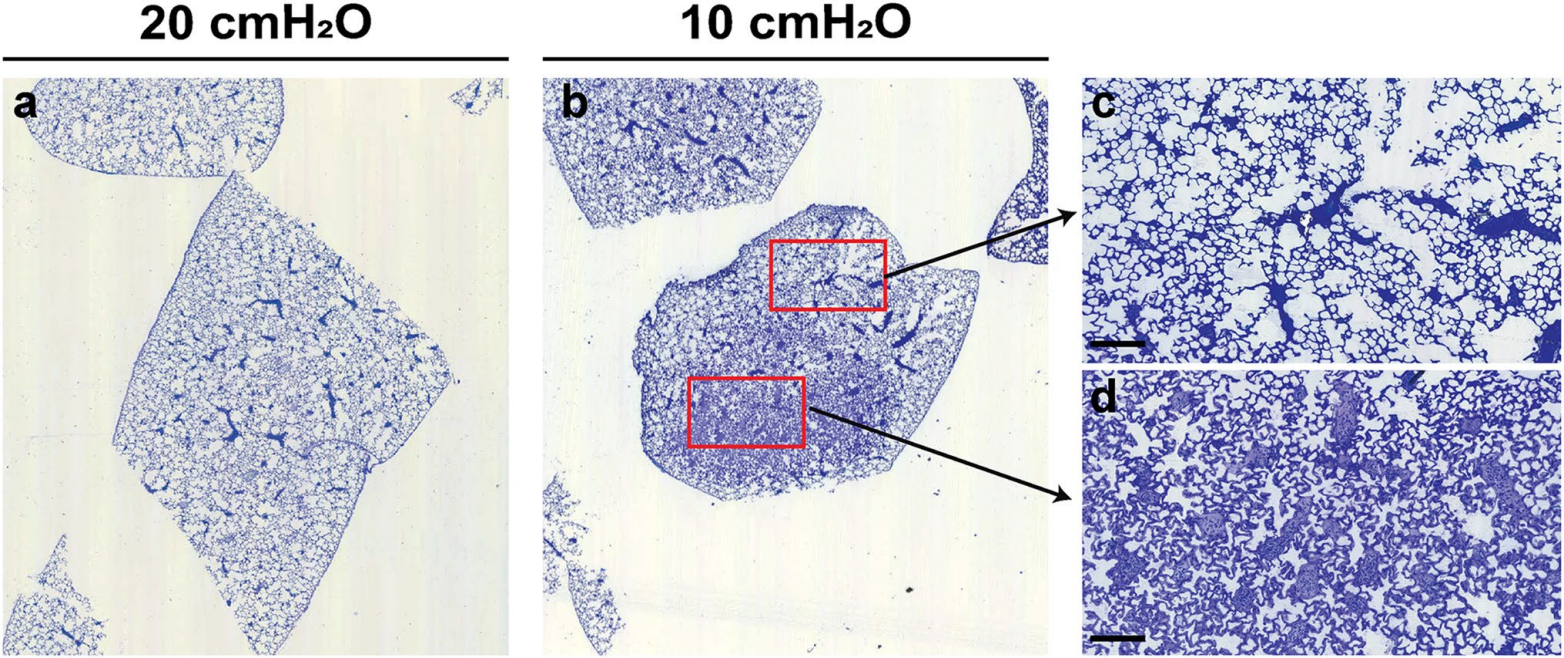
Heterogeneity in distal lung structure at variable airway pressures. Low magnification images of lung sections after lung inflation with fixative at airway pressures of **a** 20 cmH_2_O and **b** 10 cmH_2_O are depicted. **c**, **d** Regions within (**b**) were selected (enclosed by the red frame) for presentation at higher magnification. Images are representative of trends overserved in four other lungs per experimental group. A panel including an image of a lung section after fixation inflation at 30 cmH_2_O is included in Fig. S1. Scale bar: 200 µm

Visual inspection of plastic-embedded lung tissue stained with Richardson’s stain suggested that the alveolar density was highest in the 10 cmH_2_O group and lowest in the 30 cmH_2_O group (Fig. 2, *left-hand* column of images). Additionally, the septa appeared thicker in the 10 cmH_2_O group, compared with the 20 cmH_2_O and 30 cmH_2_O groups (Fig. 2, *right-hand* column of images). Examination of lung tissue sections at low magnification revealed a comparable pattern of uniform inflation in the 20 cmH_2_O and 30 cmH_2_O groups (Fig. S1). However, comparison of low-magnification images of lung tissue sections from the 20 cmH_2_O group (Fig. 3a) and 10 cmH_2_O group (Fig. 3b), revealed a heterogeneous pattern of inflation in the 10 cmH_2_O group (compare Fig. 3c and d), where the detection of bridges was complicated by the compactness of the tissue in some regions of the lungs. This challenge presented by the 10 cmH_2_O group is further evident in Fig. S2, where increasing the coverage [either from 3 to 10% (Fig. S2a, b) or from 3 to 6% (Fig. S2c–f)] markedly affected estimates of all stereological parameters. Collectively, these data suggest that a *P*_aw_ of 10 cmH_2_O is insufficient to properly inflate the distal lung, and that a *P*_aw_ of 30 cmH_2_O distends the lung structure by over-inflation.

**Fig. 4:**
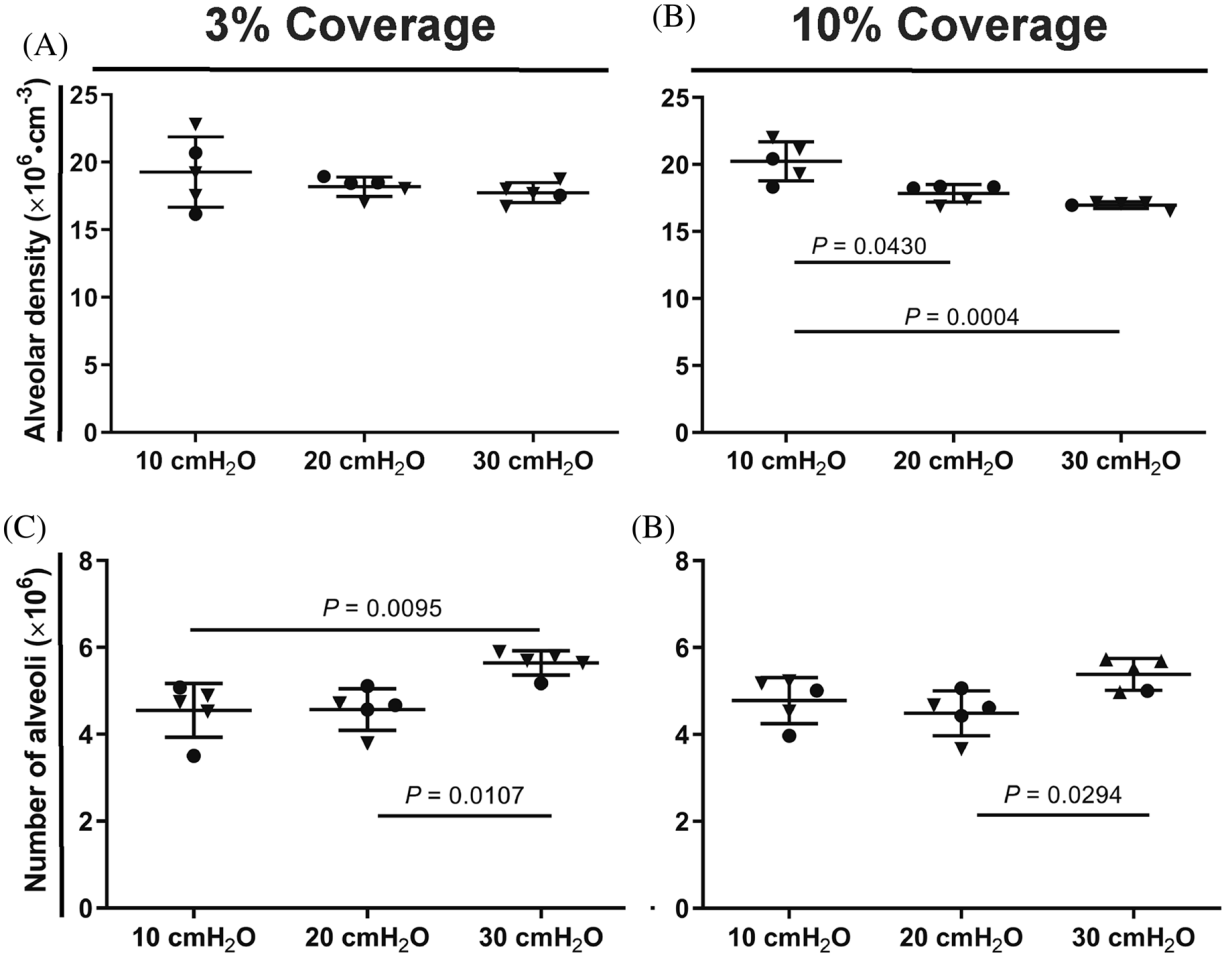
Quantitative estimation of alveoli by design-based stereology. The **a**, **b** alveolar density and **c**, **d** number of alveoli per lung were estimated using design-based stereology at 3% and 10% coverage of the region of interest, after lung inflation-fixation at 10, 20, and 30 cmH_2_O. Data reflect mean ± S.D. (*n* = 5 lungs per experimental group). Closed inverted triangles denote female animals, closed circles denote male animals. Data comparisons were performed by one-way ANOVA with Tukey’s post hoc test. Data comparisons with *P* < 0.05 are indicated. Additional stereology parameters are presented in Tables 1 and 2

Using design-based stereology to estimate parameters that describe the distal lung architecture, the alveolar density comparing the 10 cmH_2_O, 20 cmH_2_O, and 30 cmH_2_O groups was similar at 3% coverage (Fig. 4a). However, this observation did not reflect the trends noted by visual inspection of lung sections from these three experimental groups (Fig. 2). For this reason, alveolar density was re-assessed at 10% coverage, where the alveolar density of the 10 cmH_2_O group was revealed to be higher than that of the 20 cmH_2_O and 30 cmH_2_O groups, most likely reflecting the heterogeneity of lung inflation in the 10 cmH_2_O group (Fig. 3). The alveolar density comparing the 20 cmH_2_O and 30 cmH_2_O groups was unchanged irrespective of 3 or 10% coverage (Fig. 4a, b). However, a higher number of alveoli was estimated in lungs inflated at 30 cmH_2_O, most likely due to the increased volume estimated for those lungs (Fig. 1e), due to distension of the lungs at a *P*_aw_ of 30 cmH_2_O.

**Fig. 5:**
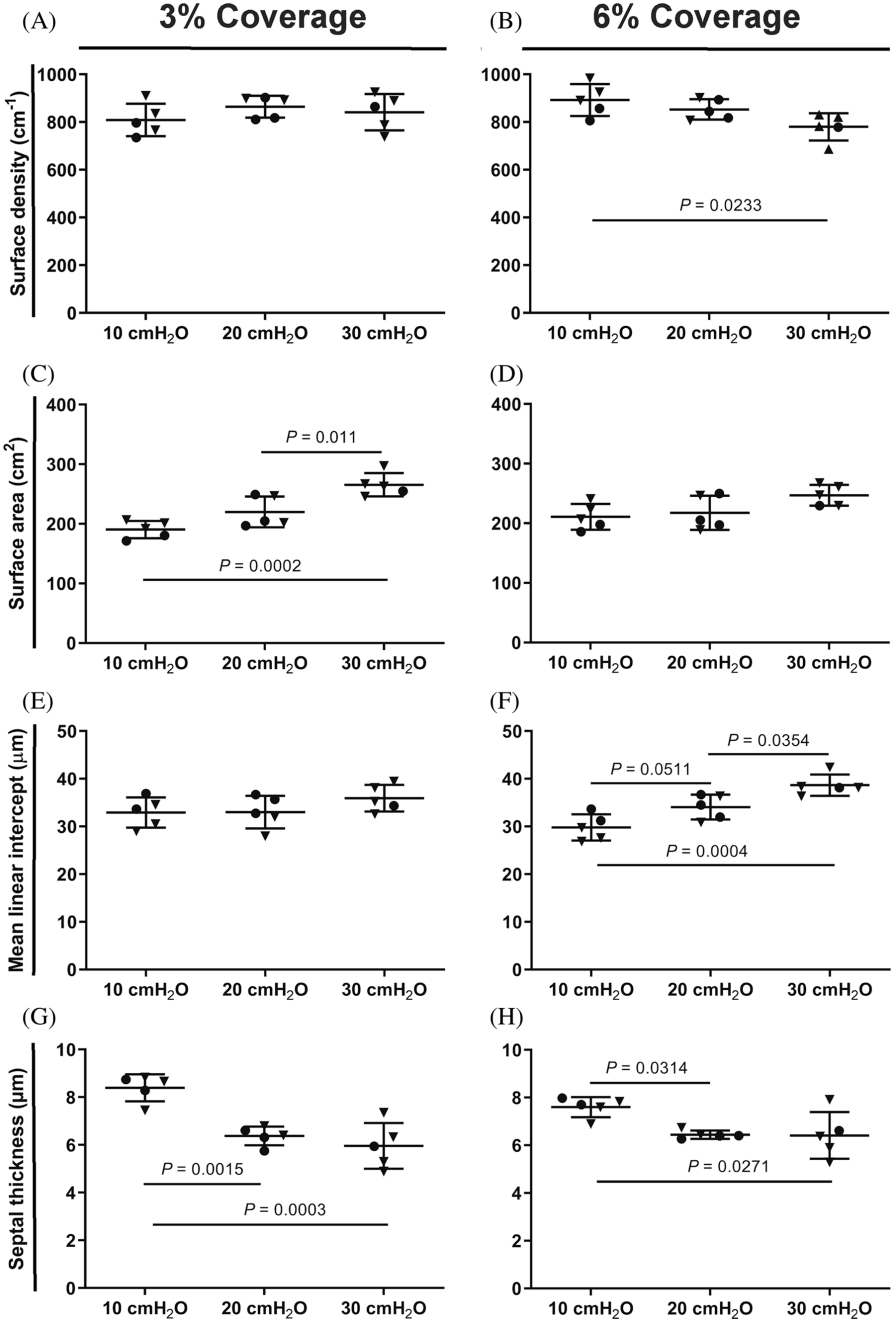
Quantitative estimation of the distal lung architecture by design-based stereology. The **a**, **b** surface density, **c**, **d** gas-exchange surface area, **e**, **f** mean linear intercept, **g**, **h** arithmetic mean septal thickness were estimated using a design-based stereology approach using 3 and 6% coverage of the region of interest, after lung inflation-fixation at 10, 20, and 30 cmH_2_O. Data reflect mean ± S.D. (*n* = 5 lungs per experimental group). Closed inverted triangles denote female animals, closed circles denote male animals. Data comparisons were performed by one-way ANOVA with Tukey’s post hoc test. Data comparisons with *P* values < 0.05 are indicated. Additional stereology parameters are presented in Tables 1 and 2

Increasing the coverage of the regions of interest was instructive for a number of other parameters that describe the distal lung structure, where no differences in surface density were noted comparing the 10 cmH_2_O, 20 cmH_2_O, and 30 cmH_2_O groups assessed at 3% coverage (Fig. 5a). However, increasing coverage to 6% revealed a decreased surface density in the 30 cmH_2_O group, which is consistent with lung distension in the 30 cmH_2_O group (Fig. 5b). The increased lung volume estimated for the 30 cmH_2_O group also impacted other volume-dependent parameters, such as gas-exchange surface area (which relates surface density to lung volume). At 3% coverage, while the estimated surface density for the 10 cmH_2_O, 20 cmH_2_O, and 30 cmH_2_O groups was constant, a higher gas-exchange surface area was estimated for the 30 cmH_2_O group, again reflecting lung distension. At 6% coverage, the magnitude of the gas-exchange surface area of the 30 cmH_2_O group was still numerically greater—but not statistically significantly different from—the 10 cmH_2_O and 20 cmH_2_O groups (Fig. 5d; Table 2), which is attributable to a lower surface density having been estimated for the 30 cmH_2_O group at 6% coverage (Fig. 5b). Increasing coverage from 3 to 6% also highlighted an increased MLI in the 30 cmH_2_O group compared with the 20 cmH_2_O group, which reflects the distension of the lungs in the 30 cmH_2_O group. Similarly, at 6% coverage, the magnitude of the MLI was numerically lower in the 10 cmH_2_O group compared with the 20 cmH_2_O group (which approached statistical significance, *P* = 0.0511; Fig. 5f), indicating underinflation of the lungs in the 10 cmH_2_O group. That the lungs in the 10 cmH_2_O group were underinflated is also supported by the increased arithmetic mean septal thickness noted at both 3 and 6% coverage in the lungs of the 10 cmH_2_O group compared with the 20 cmH_2_O and 30 cmH_2_O groups (Fig. 5g, h).

The objective of the present study was to determine the optimal *P*_aw_ for the instillation fixation of developing mouse lungs. The data presented here suggest that low *P*_aw_ (of 10 cmH_2_O) results in heterogeneous (under)inflation of lungs, where better and worse inflated areas confound stereological analyses. In contrast, high *P*_aw_ (of 30 cmH_2_O) leads to distortion of the lung volume by overinflation, leading to errors in the estimation of volume-dependent parameters, such as a total number of alveoli and gas-exchange surface area. As such, a *P*_aw_ of 20 cmH_2_O emerged as an optimal *P*_aw_ for instillation fixation, which is supported by the consistently low variance in values of stereology parameters at a *P*_aw_ of 20 cmH_2_O [comparing 3% *versus* 10% (Fig. 4) or 3% *versus* 6% (Fig. 5) coverage], compared to the 10 cmH_2_O and 30 cmH_2_O groups (summarized in Fig. S2). As such, based on the data presented here, a *P*_aw_ of 20 cmH_2_O is recommended for the inflation fixation of the lungs of mouse pups.

By way of caveats, it is important to recognize that healthy lung tissue was employed in the present study, and thus, all three experimental groups would exhibit comparable elastic recoil. However, in the extension of these studies to the analysis of diseased lung tissue, where the parenchymal or elastin fraction of the tissue may be different between control and experimental groups, differences in elastic recoil between the groups is to be expected. This might affect the behavior of the lungs during and after instillation fixation; and during fixation, the retraction of lung tissue (und thus, lung volume) may occur to different degrees between control and experimental groups. This may be controlled for by performing Cavalieri estimates of lung volume before and after embedding.

## Supplementary Information

Below is the link to the electronic supplementary material.

Supplementary file1 (DOCX 31 KB)

Supplementary file2 (TIF 12935 KB)

Supplementary file3 (TIF 1551 KB)
