# Supplementary material for: A comparison of airway pressures for inflation fixation of developing mouse lungs for stereological analyses
Source: Histochem Cell Biol. 2020 Dec 29;155(2):203–14. doi: 10.1007/s00418-020-01951-0 (PMC7910376; doi:10.1007/s00418-020-01951-0)
Supplement: Supplementary file 1 — Supplementary file1 (DOCX 31 KB) [file 418_2020_1951_MOESM1_ESM.docx]

Online supplement for:

A comparison of airway pressures for inflation fixation of developing mouse lungs for stereological analyses

David Pérez-Bravo^1,2^ · Despoina Myti^1,2^ · Ivana Mižíková^1,2,3,4^ · Tilman Pfeffer^1,2,5^ · David E. Surate Solaligue^1,2,6^ · Claudio Nardiello^1,2^ · István Vadász^2,7^ · Susanne Herold^2,7^ · Werner Seeger^1,2,7,8^ · Katrin Ahlbrecht^1,2^ and Rory E. Morty^1,2,7^

1 Department of Lung Development and Remodelling, Max Planck Institute for Heart and Lung Research, member of the German Center for Lung Research (DZL), Parkstrasse 1, 60231 Germany

2 Department of Internal Medicine (Pulmonology), University of Giessen and Marburg Lung Center (UGMLC), member of the German Center for Lung Research (DZL), Aulweg 123, 35394 Giessen, Germany

3 Regenerative Medicine Program, The Ottawa Hospital Research Institute, 501 Smyth (Box 511), Ottawa, Ontario, 1H 8L6, Canada

4 Department of Cellular and Molecular Medicine, University of Ottawa, 451 Smyth Road, Ottawa, Ontario, K1H 8M5, Canada

5 Centre for Paediatric and Adolescent Medicine, Im Neuenheimer Feld 430, 69120 Heidelberg, Heidelberg University Hospital, Heidelberg, Germany

6 Our Lady's Hospital, Moathill, Navan, Co. Meath, C15 RK7Y, Ireland

7 Cardio Pulmonary Institute, Justus Liebig University Giessen, Klinikstrasse 33, Giessen, Germany

8 Institute for Lung Health (ILH), Justus Liebig University Giessen, Aulweg 130, Giessen, Germany

This online supplement consists of two supplementary figures: Fig. S1 and Fig. S2, and their associated figure legends:

**Fig. S1**  Heterogeneity in distal lung structure at variable airway pressures. Low magnification images of lung sections after lung inflation with fixative at airway pressures of (**a**) 10 cmH_2_O, (**b**) 20 cmH_2_O, and (**c**) 30 cmH_2_O are depicted. Images are representative of trends overserved in four other lungs per experimental group. Panels (a) and (b) are the same images depicted in Fig. 3 in the manuscript proper. Scale bar: 1 mm

**Fig. S2**  Variance in the mean values for stereologically-determined parameters that describe the lung structure assessed with low *versus* high percentage coverage of the regions of interest. To determine the variance in the mean values, the absolute difference between mean values in three different inflation fixation airway pressure groups was calculated, by subtracting the high percentage coverage from the low percentage coverage within the same *P*_aw_ group. This variance reveals how much the percentage coverage affects the mean values for stereological parameters determined at the same inflation fixation *P*_aw_. The variance in (**a**) alveolar density comparing 3% *versus* 6% coverage (Fig. 4a *versus* Fig. 4b), (**b**) number of alveoli comparing 3% *versus* 6% coverage (Fig. 4c *versus* Fig. 4d), (**c**) gas-exchange surface area comparing 3% *versus* 6% coverage (Fig. 5c *versus* Fig. 5d), (**d**) surface density comparing 3% *versus* 6% coverage (Fig. 5a *versus* Fig. 5b), (**e**) arithmetic mean septal thickness comparing 3% *versus* 6% coverage (Fig. 5g *versus* Fig. 5h), and (**f**) mean linear intercept comparing 3% *versus* 6% coverage (Fig. 5e *versus* Fig. 5f)
