# Supplementary figures and images for: A comparison of airway pressures for inflation fixation of developing mouse lungs for stereological analyses
Source: Histochem Cell Biol. 2020 Dec 29;155(2):203–14. doi: 10.1007/s00418-020-01951-0 (PMC7910376; doi:10.1007/s00418-020-01951-0)

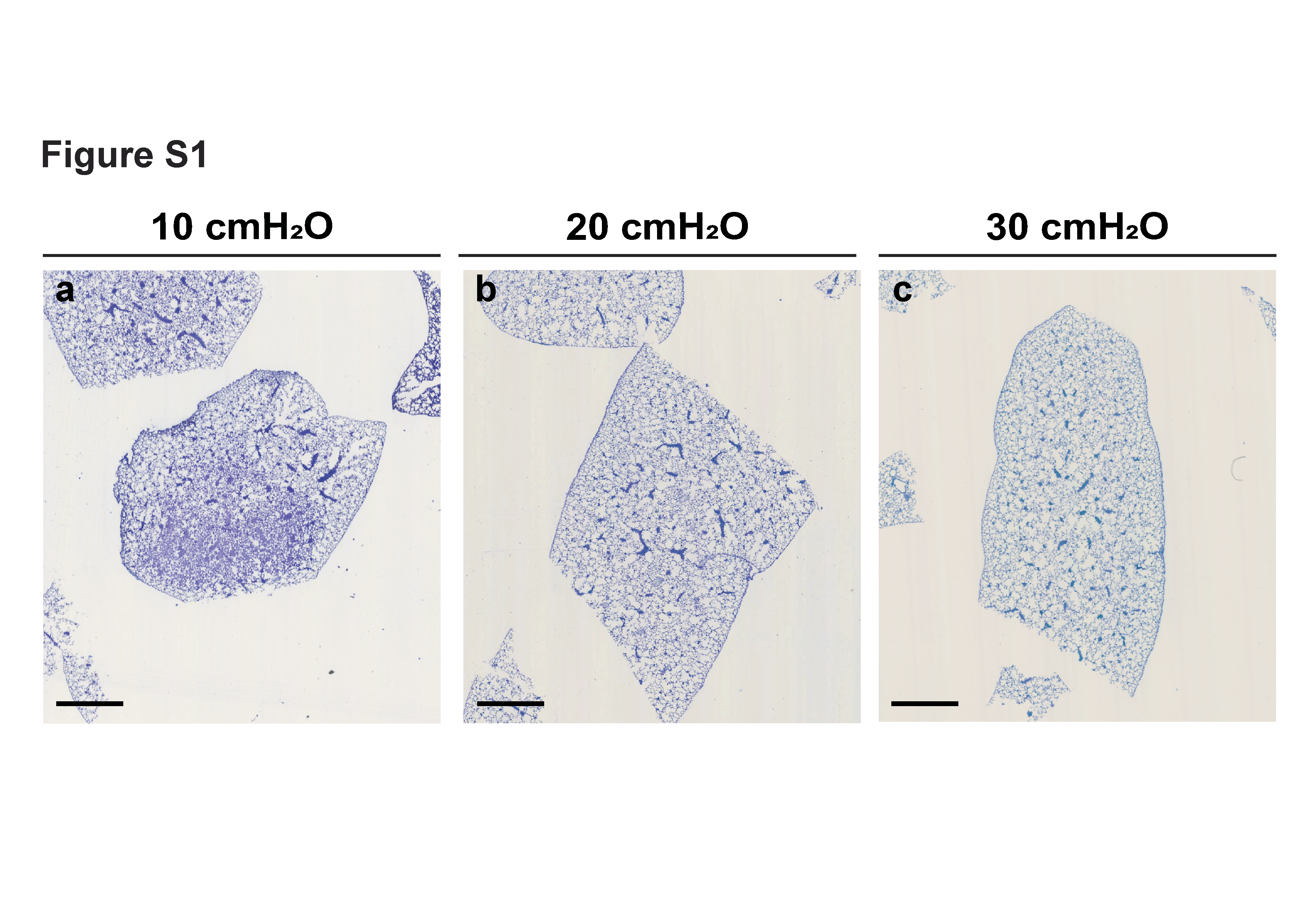

Supplement: Supplementary file 2 — Supplementary file2 (TIF 12935 KB) [file 418_2020_1951_MOESM2_ESM.tif]

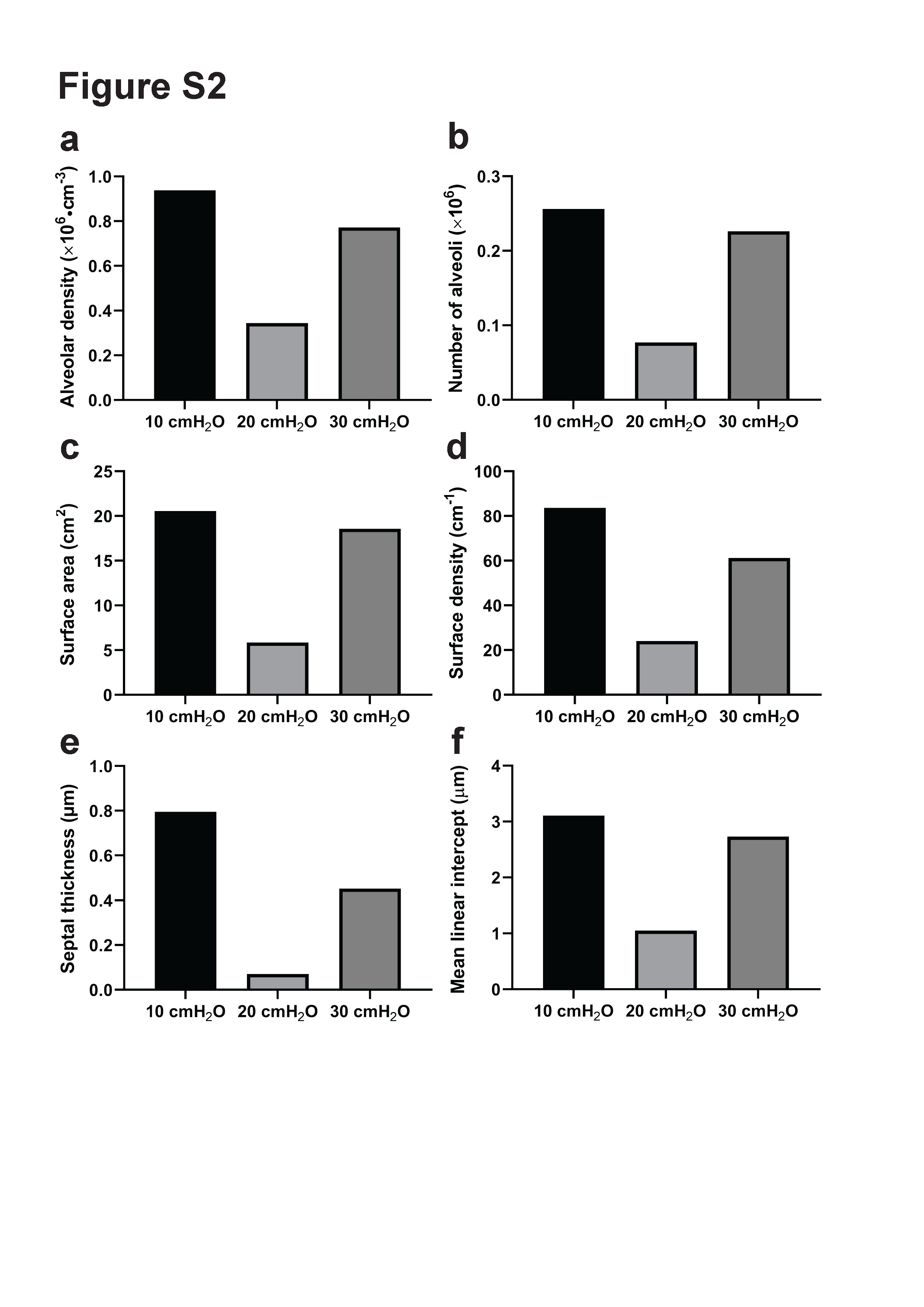

Supplement: Supplementary file 3 — Supplementary file3 (TIF 1551 KB) [file 418_2020_1951_MOESM3_ESM.tif]
